# Personality and Insomnia Symptoms in Older Adults: The Baltimore Longitudinal Study of Aging

**DOI:** 10.1093/geroni/igaa057.1925

**Published:** 2020-12-16

**Authors:** Darlynn Rojo-Wissar, Amal Wanigatunga, Eleanor Simonsick, Antonio Terracciano, Jennifer Schrack, Sharmin Hossain, Paul Costa, Adam Spira

**Affiliations:** 1 Johns Hopkins Bloomberg School of Public Health, Baltimore, Maryland, United States; 2 National Institute on Aging, Bethesda, Maryland, United States; 3 Florida State University College of Medicine, Tallahassee, Florida, United States; 4 Duke University School of Medicine, Durham, North Carolina, United States

## Abstract

Personality and disturbed sleep are tied to medical morbidity in older adults. We examined associations of personality dimensions and facets from the five-factor model with reports of insomnia symptoms in 1,069 well-functioning older adults 60-97 (SD=8.64) years (51% women) from the Baltimore Longitudinal Study of Aging. Personality was assessed by the Revised NEO Personality Inventory, and insomnia symptoms measured by the Women’s Health Initiative Insomnia Rating Scale. Adjusting for demographics and depressive symptoms, higher neuroticism (B=0.05, SE=-0.01, p<.001) and lower conscientiousness (B=-0.03, SE=-0.01, p<.05) were associated with greater insomnia severity. Although openness, extraversion and agreeableness were not associated with insomnia, a facet of each was. Higher scores on the “positive emotions” facet of extraversion (B =-0.03, SE=-0.01, p<.05) “ideas” facet of openness (B=-0.03, SE=-0.01, p<.05) and altruism facet of agreeableness (B=-0.03, SE=-0.01, p<.05) were associated with lower insomnia severity. Sleep disturbances may partially mediate personality’s influence on health. Part of a symposium sponsored by the Sleep, Circadian Rhythms and Aging Interest Group.

